# Formative Development of a Weight Management Intervention for Adolescents with Type 1 Diabetes Mellitus and Obesity

**DOI:** 10.1155/2023/9584419

**Published:** 2023-04-19

**Authors:** Jennifer Warnick, Katherine E. Darling, Lisa Swartz Topor, Elissa Jelalian

**Affiliations:** 1 Alpert Medical School of Brown University, Providence, USA; 2 The Miriam Hospital, Providence, USA

## Abstract

The prevalence of overweight and obesity in youth with type 1 diabetes mellitus (T1D) now exceeds that of youth without T1D. Comorbid T1D and excess adiposity are associated with multiple serious negative health outcomes. Unfortunately, youth with T1D are often excluded from and/or not referred to standard behavioral lifestyle interventions. This is often attributed to the complexities of managing T1D and an effort not to overburden persons who have T1D. Furthermore, standard behavioral weight management intervention recommendations can be perceived as contradicting T1D disease management (e.g., removing sugar-sweetened beverages from diet, energy balance with exercise, and caloric restriction). A weight management intervention specifically designed for youth with T1D is needed to provide treatment to youth with comorbid T1D and overweight/obesity. The current study interviewed adolescents with T1D and overweight/obesity (*n* = 12), their caregivers (*n* = 12), and pediatric endocrinologists (*n* = 9) to understand (a) whether they would be interested in a weight management intervention adapted for youth with T1D and (b) specific adaptations they would want and need. Five central themes emerged following applied thematic analysis: (1) program content, (2) programmatic messaging, (3) program structure, (4) social support, and (5) eating disorder risk. Results provide detailed recommendations for the adaptation of a behavioral weight management intervention for youth with T1D.

## 1. Introduction

Obesity in youth with type 1 diabetes mellitus (T1D) is a major public health problem as the prevalence of overweight/obesity in this population has risen exponentially over the past 30 years [1-4]. The prevalence of overweight and obesity in youth with T1D now exceeds that of youth without T1D, affecting 40% of all adolescents with T1D and nearly half of the girls with T1D [4, 5]. T1D and obesity are independently associated with increased risks for cardiovascular disease events, thus youth with *both* T1D and obesity are at compounded risks for morbidity and early mortality [6, 7]. Furthermore, youth with comorbid T1D and obesity are developing insulin resistance to their exogeneous insulin. This leads to a cycle of weight gain and increased insulin requirements which, once set in motion, is difficult to change as obesity is likely to continue into adulthood especially in the T1D population [8, 9].

Adolescence is a vulnerable age group among youth with T1D. Adolescents tend to experience rapid weight gain around puberty and are transitioning to more autonomous eating and T1D management [10-12]. Adolescents with T1D have the highest average hemoglobin A1C (HbA1C) across the lifespan compared to other age groups due, in large part, to these developmental changes in their physical and psychosocial functioning [12, 13]. Adolescents with excess weight are also at high risk for experiencing negative psychosocial outcomes, such as depression, weight-related bullying, anxiety, and decreased quality of life [14-16]. Taken together, immediate intervention is needed to adequately treat adolescents with excess weight and T1D.

Behavioral lifestyle interventions (BLIs) are evidence-based treatments for adolescent obesity that show short- and long-term effectiveness in reducing weight status among adolescents with excess weight [17-20]. Guidelines from the US Preventive Services Task Force (USPSTF) and the American Academy of Pediatrics (AAP) suggest high-intensity BLIs for the treatment of adolescent obesity [21, 22]. BLIs can be structured in multiple ways to meet these guidelines. Often, evidence-based BLIs for adolescents are structured with weekly group-based sessions for approximately 16 weeks, followed by tapered maintenance sessions for a 6- or 12-months total length of treatment [17, 18, 20, 21, 23]. Other models for BLIs can include either individual sessions or hybrid models combining group and individual sessions [22]. Session content typically focuses on a combination of psychoeducation and behavioral strategies to emphasize core concepts such as self-monitoring, balanced nutrition, stimulus control, physical activities, and goal setting. Adolescents receive an individualized caloric deficit diet goal. There is also a focus on gradual increase in physical activity, and adolescents are encouraged to self-monitor their diet and activity. Caregivers typically attend periodic parallel caregiver sessions [24, 25].

There are unique aspects to T1D management that can impede engagement in standard BLIs for youth [26-28]. Some of these difficulties include (1) dysregulated hunger/satiety signals due to fluctuating blood glucose levels, (2) blood glucose changes associated with physical activity, and (3) eating due to reasons other than hunger (i.e., fear of hypoglycemia, to treat lows, and in preparation for physical activity), and (4) heightened risks for disordered eating attitudes and behaviors unique to T1D [26-28]. Episodes of hypoglycemia are often associated with intense hunger and require consumption of fast-acting carbohydrates (e.g., juice and candy) [27]. In BLIs, these types of energy-dense/nutrient-deficient foods are discouraged, and many BLIs suggest removing them from the home environment using stimulus control principles [29]. Similarly, youth with T1D and/or their caregivers may experience fears of hypoglycemia, particularly at bedtime, and purposefully consume excess snacks to keep blood glucose levels elevated to avoid lows [28], leading to regular excess calorie consumption.

Engagement in physical activity can also be a barrier for adolescents with T1D, given the impact of physical activity on blood glucose stability. In previous qualitative research, adolescents with T1D expressed concerns related to the potential for hypoglycemic episodes during and/or following exercise bursts [28]. Persons with T1D often need to eat additional snacks *prior* to exercising to prevent hypoglycemic episodes. Furthermore, episodes of hypoglycemia during physical activity can require necessary breaks in activity (i.e., immediately stopping of activity and resting for a minimum of 15 minutes) and require excess energy consumption to adjust dropping blood glucose levels. This can, understandably, cause frustration and feel counter-productive with regards to “energy balance” education provided in standard BLIs [28].

The prevalence of disordered eating symptoms is greater amongst adolescents with T1D (40%) compared to peers without T1D (30%), and approximately 7% of adolescents with T1D have been diagnosed with an eating disorder as compared to 2.8% of those without T1D [30-32]. One such type of disordered eating behavior specific to T1D is insulin purging, a dangerous behavior where persons with T1D will purposefully omit necessary insulin administration to induce glycosuria [30]. Known by some as “diabulimia,” it is estimated that 20–40% of youth with T1D have used insulin manipulation for weight loss [30, 33]. Although evidence discredits concerns regarding the impact of structured BLIs on disordered eating behaviors [34, 35]; adolescents with T1D have unique risk factors for developing disordered eating behaviors that need to be thoughtfully considered when engaging this population in any type of weight management intervention.

Despite the clear need for BLIs focused on adolescents with T1D, there is a paucity of evidence-based treatment recommendations and programs for youth with comorbid overweight/obesity and T1D. In 1993, Thomas-Dobersen et al. evaluated an adapted BLI for adolescents with T1D in a small pilot study [36]. The intervention produced a 3% weight reduction in the intervention group compared to a 0.9% weight gain in the control group; however, the sample size was small and homogeneous (*n* = 20; 95% female), and groups were not randomized. Prior research has found that adults with T1D and obesity who participated in a BLI for adults with diabetes demonstrated significantly reduced body weight, HbA1C, and total insulin doses prescribed compared to a matched usual care control group [37]. Nonetheless, there is a preliminary signal that an adapted adolescent BLI for T1D is feasible and potentially effective, yet further research is needed to develop such a program.

Collecting participant feedback in the development of interventions is an essential procedure to ensure acceptability and feasibility [38]. This is particularly important for novel interventions. Even if an intervention is well supported by science, does not guarantee the target patient population will be encouraged to initiate and continue participation. Qualitative interviews to inform intervention development are a useful strategy to ensure initial acceptability [39]. The goal of the present study was to determine intervention adaptations needed to create a BLI specific to adolescents with T1D (BLI + T1D) from the perception of adolescents, caregivers, and endocrinologists.

## 2. Methods

### 2.1. Participants.

Participants included adolescents with T1D and overweight/obesity, caregivers of these adolescents, and pediatric endocrinologists. All participants were recruited from the New England area. Adolescents were eligible if they were between the ages of 13–17 years, diagnosed with T1D at least six months prior to study enrollment, and had a body mass index (BMI) equal to or greater than the 85th percentile for sex and age. Eligible caregivers were the legal guardians of adolescents with comorbid T1D and overweight/obesity. Pediatric endocrinologists were eligible if they were currently providing medical care to adolescents with T1D. All eligible participants were English-speaking. Recruitment occurred between January 2021 and August 2021. Adolescents and caregivers were recruited through flyers distributed in local pediatric endocrinology offices, physician referrals, and letters mailed to potentially eligible participants identified through the electronic medical record. Participating pediatric endocrinologists were recruited via phone calls and emails to pediatric endocrinology offices in New England.

### 2.2. Procedures.

A qualitative research design was chosen to capture in-depth thoughts, opinions, and suggestions related to the development of BLI + T1D for adolescents. In addition, participants completed brief quantitative measures, and study staff pulled adolescents’ relevant electronic medical record information if permission was granted. After consenting, participants completed a remote individual interview with a study staff member. Interviews lasted approximately one hour. Following the interview, participants completed quantitative measures. Each participant received an e-gift card for completing the study. All study procedures were approved by the hospital’s Institutional Review Board.

### 2.3. Measures

#### 2.3.1. Qualitative Interviews.

Three separate semistructured interview guides were used, tailored to each group of participants (i.e., adolescents, caregivers, and endocrinologists); however, primary concepts covered were consistent between participant groups. Initial interview guides were created by the first author, and the two senior authors on this paper (i.e., a clinical psychologist with expertise in BLIs and a pediatric endocrinologist) provided feedback. Their feedback resulted in a revised, final version of the three guides. Questions to adolescents and caregivers inquired about knowledge of current national guidelines for healthy living, perception of teen’s personal engagement in these behaviors, interest in change, any ways in which diabetes affects engagement in healthy living behaviors or vice versa, and whether there has ever been concern about weight or body shape. Endocrinologists were asked how they typically manage comorbid T1D and overweight/obesity with their patients; discussion they have with their patients regarding healthy lifestyle behaviors, weight, and managing mood concerns; the structure of their clinic (i.e., other multidisciplinary professionals present); and their perceived prevalence and treatment of comorbid eating disorders among patients with T1D. All participants (i.e., adolescents, caregivers, and endocrinologists) were asked about their interests in a future program for BLI + T1D and sought feedback on program design considerations. Semistructured questions served as a guide allowing the interviewer to ask follow-up questions to clarify responses and gather additional detail and explanation.

Two authors met regularly throughout the data collection process to discuss initial observations and data saturation. Interviews were conducted until both authors agreed data saturation was met. All interviews were audio-recorded and transcribed verbatim.

#### 2.3.2. Quantitative Measures.

Following the interview, caregivers and endocrinologists completed a brief demographics survey, and adolescents completed the following validated self-report measures. Participants were asked to identify their biological sex assigned at birth as well as their current gender identity. Gender identity is presented in the results; biological sex assigned at birth was used to calculate BMI and BMI percentiles.

The Diabetes Eating Problems-Survey, Revised (DEPS-R) is a 16-item diabetes-specific self-report measure of disordered eating in patients with T1D [40]. Statements about attitudes and behaviors related to diabetes management and eating/weight-related cognitions and behaviors are rated on a 6-point Likert scale (0 = *never* to 5 = *always*), with higher scores indicating more disordered eating symptoms. The DEPS-R has demonstrated excellent internal consistency and external validity amongst youth with T1D [40]. Possible total scores can range from zero to 80. Though there is no clinical cutoff for disordered eating using this measure, a score ≥20 is correlated with higher risk for diabetes complications, higher HbA1C, and higher risk for disordered eating diagnosis [40, 41].

The problem areas in diabetes, teen version (PAID-T) is a 26-item measures of diabetes-specific emotional distress in teens with T1D [42]. Participants rate how much each item applies to them over the past month using a 6-point Likert scale (1 = *not a problem* to 6 = *serious problem*). The measure is both reliable and valid for use with adolescents with T1D [43]. Possible total scores can range from 14 to 84, with higher scores indicating greater diabetes-related distress, and with scores ≥44 indicating clinically significant diabetic distress [43].

Participants also provided consent to collect the following electronic medical record data, if available: date of diagnosis, medication list, most recent HbA1C, most recent percent time in target blood glucose range (i.e., 70–180 mg/dL), number of endocrinology visits in the past 5 years, and most recently measured BMI.

### 2.4. Data Analysis.

All interviews were transcribed and deidentified. Applied thematic analysis was used for data analysis [44]. Through this approach, an initial coding structure reflecting semistructured interview prompts was developed. Two independent coders (JW and KD) familiarized themselves with the data (i.e., read transcripts) and refined the coding structure. Following coding structure refinement, each transcript was coded independently by both coders. Coders met regularly to discuss and clarify discrepancies for each transcript until coders reached 100% agreement on all codes. Emerging themes were identified by coders during this process. Final codes were entered into NVivo [45]. Data were then iteratively reduced through consultation among authors focused on identifying themes and subthemes. The descriptive analysis was used to examine the demographic and self-report measure data using IBM SPSS Statistics (Version 26).

## 3. Results

### 3.1. Participants.

Participants included 12 adolescents with T1D, 12 caregivers, and 9 pediatric endocrinologists (see Table 1 for demographic information). The average BMI percentile for adolescents was 96.42 (*SD* = 2.27), and 10 out of 12 adolescent participants met criteria for obesity. Most adolescent participants used continuous glucose monitors (CGMs; 83.3%), with 75% using pumps and 25% used multiple daily injections for insulin needs. Seven adolescents had CGM data for the 14 days prior to data collection; of those with CGMs, average time in range was 57.43% (*SD* = 18.85). Adolescents had attended an average of 2.2 (*SD* = 1) endocrinology appointments per year for the past 5 years. The average PAID-T score was 36.83 (*SD* = 15.2; *range* = 16–65; 25% had a total score ≥44). The average DEPS-R score was 22.50 (*SD* = 8.6; range = 5–33; 66.7% had a total score ≥20).

### 3.2. Qualitative Analysis.

Overall, participants indicated interest and were eager to learn about a BLI + T1D program. Beyond initial interest, participants reflected on programmatic aspects that they wanted to see included. Interviews were open-ended, with participants not receiving information on standard BLIs for adolescents; however, many components suggested were consistent with standard adolescent BLIs and/or extended standard BLI content to better fit the needs specific to youth with T1D. The following themes were identified: (1) program content, (2) programmatic messaging, (3) program structure, (4) social support, and (5) eating disorder risk. The following describes each theme, providing quotes from participants to illustrate each theme. Table 2 provides additional quotes from participants organized by themes.

### 3.3. Program Content.

Participants suggested numerous topics to be included in a BLI + T1D that are typically discussed in a standard BLI. These topics included healthy nutrition and exercise education, weight loss guidance, and building a positive body image. Regarding general healthy nutrition and exercise, one teen stated, “*I would be interested in getting help from a dietician or someone who can help me with some exercise*” (17 years, male) and another (14 years, female) was interested in “*how to stay fit and healthy in general*.” Similarly, one caregiver requested “*specific tips we could all try to change our lifestyles*…*without making it overly burdensome*” (male).

Many participants identified the lack of healthy lifestyle resources specific to persons with T1D. One teen explained that she looked into a commercial weight loss program, but then “*realized if I did it, I would die. If a diabetic were to follow that type of program*… *it would be very detrimental and dangerous for us*” (14 years, female). Participants requested education on safe ways to improve their nutrition, physical activity, and weight while managing T1D. Specifically, they asked for where to find accurate information, challenges of weight loss in T1D, exercise while avoiding hypoglycemic events, and T1D-specific nutrition suggestions.

Along the same lines, many endocrinologists wanted participants in a BLI + T1D to learn how healthy lifestyle behaviors and T1D management are bidirectionally affected. One endocrinologist (male) stated “*people with diabetes are most concerned about their A1C*, *and they get A1C*, *and thus talking about improving exercise and eating can be framed in a way that promotes improving A1C and insulin sensitivity. You are motivating them with something very objective*.”

Participants also wanted a BLI + T1D to provide education and skills related to mental health issues common to those with T1D, including diabetes burnout, social anxiety, and normalization of T1D. One teen said, “*I feel like people should be more aware of diabetes burnout. I did not know until recently that was a thing. I thought it was just me*” (15 years, female). Caregivers also requested the program include content about mental health. One caregiver (female) requested “*providing caregivers with information on what to look for regarding mental health concerns and the importance of how mental health plays into physical health*.” Many participants wanted some topics to be included that had nothing to do with T1D directly, even though they knew the entire group would consist of youth with diabetes. Teen 12 (14 years, female) explained “*there is so much about diabetes in our lives. Sometimes it is nice to have something catered towards diabetes without it directly being like,* “*yeah, you have diabetes, do not forget*.”

### 3.4. Programmatic Messaging.

Participants expressed mixed feelings about discussing weight directly. Many caregivers and endocrinologists expressed hesitation about addressing weight directly with adolescents. Caregivers explained that it may be an uncomfortable topic, while endocrinologists had concerns that mentioning weight might cause eating disorders or overwhelm adolescents who have a chronic illness. On the other hand, many adolescents stated they were interested in receiving information and having discussion about weight loss. In fact, 83% of adolescents endorsed “losing weight is important to me” on quantitative measures (DEPS-R). One teen (17 years, female) said “*I think it would be very important to talk about weight loss. I know a lot of people struggle with weight loss. It is hard and I have struggled big time. You try everything, absolutely everything*, *and nothing helps*.” Another (15 years, female) stated “*I do not think it (weight) should be avoided because I know some girls want to talk to somebody about it, but they do not know who to approach*.” Follow-up discussion with the caregivers who were hesitant to discuss weight directly in a BLI + TID determined that they would be more comfortable if the program discussed positive body image and that the messaging around weight was important.

Regarding general program messaging, adolescent participants made it clear they wanted the program to avoid being too directive. For example, participants wanted the messaging to encourage “opportunities” and “suggestions” for health behavior changes; they did not want to be told directly what to do. One teen explained, “*Do not tell us what not to do*… *because if you tell a teenager not to do something, they will go and do it anyways because they are like oh, I will just test the limits*” (15 years, female).

Participants stated the importance of the program leader(s) *really understanding diabetes*, with one endocrinologist saying, “*feeling that somebody within that program really understands whether it is the exercise piece of it and how that impacts blood sugars or the carb counting*… *that they really understand the additional factors that need to be taken into account. You get more buy-in*, *understandably*, *with what you are doing*, *knowing that people have really thought through this and how it can work for your family*” (female). Similarly, one teen (14 years, male) stated that they want program leader(s) who “*know what they are talking about*,” and a caregiver (female) included that they would want involvement of a professional who “*can give them the right information*.”

### 3.5. Program Structure.

Across all groups (i.e., adolescents, caregivers, and endocrinologists) most participants preferred a peer-group format, indicating that a group setting would make the program more approachable and fun. One teen (17 years, male) stated “*if you had a group of kids who you can relate to and you can talk to, you would be happier and healthier as a group of teenage type 1 diabetics. I think that would be a good thing*.” Most participants stated that the opportunity to meet peers in their area would likely attract families to the BLI + T1D. For example, one caregiver emphasized the importance of similar peers, “*that is a big reason why my daughter goes to diabetes camp, she feels like she belongs*” (male). Caregivers, in particular, stressed the importance of allowing teens to speak with other teens, “…*a teenager does not want answer from an adult. The peer interaction really is the most valuable*” (female). Alternatively, one teen and one endocrinologist preferred a one-on-one meeting between a teen and healthcare professional given potential anxiety related to meeting and sharing with new peers. Yet, two caregivers explicitly stated that one-on-one meetings with health professionals would feel too similar to “*another doctor’s appointment which would be a barrier in participating*” (female).

Regarding virtual versus in-person sessions, many participants acknowledged the convenience associated with virtual meetings as well as the opportunity to invite a more geographically diverse group of people who could attend the same group. On the other hand, participants reflected that in-person meetings, as opposed to virtual meetings, encourage more accountability, participation, and group cohesion. An endocrinologist (female) explained “…*virtual would provide easier access for families and reduce transportation barriers*, *but some patients struggle to use technology and do not have access*.” Many adolescents specifically preferred in-person meetings, explaining that they felt “zoomed out” after the COVID-19 pandemic. One adolescent (13 years, male) explained, “*Virtual gets boring. If you are in a zoom, there is probably something in your mind and you just want to get up and do something else*,” with another stating that virtual “*is a little less personal*” (14 years, female).

### 3.6. Social Support.

While peer-groups are a standard aspect of most adolescent BLIs, participants in the current study emphasized the importance of accessing social support from others who live with T1D. One endocrinologist (female) stated “*It is very validating*. … *It allows adolescents and families to see they are not alone*, *that others face these challenges*.” Teens wanted a place to meet peers living with T1D, to learn from others with T1D about diabetes-related tips, and to vent about T1D-related frustrations. One teen (17 years, female) explained that other peers with T1D could support each other in ways that someone without T1D may not recognize. “*I feel like personally it would be nice to know other type 1 diabetics who are like in it with me to know they are going through the same thing I would be going through*, *and to be able to rebound off each other*, *pick each other up*.”

Diabetes camp was often mentioned during qualitative interviews. Diabetes camp provides combined social, educational, and fun activities for youth with T1D. All participant groups (i.e., adolescents, caregivers, and endocrinologists) described the benefits of having a BLI + T1D that incorporated these elements, available during the schoolyear, as they encouraged diabetes- and health-related education and support. Multiple teens explained specific games they remembered playing at diabetes camps in the past. The games described did not specifically involve educational materials, but rather social activities that encouraged the campers to get to know each other and become friends.

Regarding family involvement, adolescents and caregivers preferred that caregivers be involved with a BLI + T1D but separately from adolescents. Adolescents wanted space to ask questions and speak freely with peers, while also wanting to make sure their caregivers were informed and involved with the program. One teen (14 years, female) suggested “*do not have them (caregivers) standing right next to you, looking over your shoulder, but like have them in the loop*.” Caregivers recognized their adolescents’ desire for independence, while acknowledging ongoing responsibility in supporting adolescents’ health behaviors. Caregivers also wanted to meet other caregivers locally who had a child with T1D for support, advice, and suggestions. One caregiver (male) discussed the difficulty he has had finding T1D support locally: “…*I ended up finding groups of parents on Facebook*… *but even that became a little overwhelming because you hear horror stories, you know*.” This quote exemplifies how anxiety provoking it can feel to become involved in large, national support groups where there is less moderation over the content shared. Multiple caregivers shared this sentiment, while also acknowledging how helpful national organizations (i.e., JDRF) have been in fostering support groups for caregivers of youth with T1D. Thus, participants wanted a BLI + T1D to involve elements of social support that may go beyond that of a standard BLI.

### 3.7. Eating Disorder Risk.

Finally, eating disorder concerns was a main theme that emerged, particularly among endocrinologists. Nearly, all pediatric endocrinologists spontaneously raised concerns about eating disorders in this population. These endocrinologists discussed the high risk of disordered eating behaviors, specifically insulin omission, among youth with T1D. One provider (male) stated “…*the big issue is diabulimia. I do not know how to approach that*… *My educators are so afraid that it is happening that they are not willing to discuss the topic at all. You do not want to bring it up to someone who does not know about it*.” Another endocrinologist (female) warned that talking about weight or providing any nutrition suggestions might cause an eating disorder, “*spinning it as a weight management thing would not be the way to go because I would worry very much about the diabulimia issue*.”

Even so, nearly half the endocrinologists interviewed stated that they did not view disordered eating/diabulimia as prevalent in their clinics. “*I havenot seen it as much as you would expect based on how prevalent it is in kids with type 1*” (female); “*I don’t have many of those patients. I don’t see that as a big problem in my patient population*” (male). One provider (male) explained that they talk with their patients if they suspect disordered eating symptoms, “*but I cannot say that it is often that I am convinced at the end of a conversation or following a patient for a while that (insulin omission) was actually going on in any sort of calculated way*.”

Adolescents and caregivers rarely brought up disordered eating. Adolescents, however, completed a measure of disordered eating symptoms specific to T1D (i.e., DEPS-R). Out of the 12 adolescents who completed the study, 67% reported clinically significant eating disorder scores (≥20 on DEPS-R) [40], including five females and three males. Taking a more detailed look at the questionnaire results, 60% endorsed “*when I overeat, I do not take enough insulin to cover the food,*” 92% endorsed “*I feel that it is difficult to lose weight and control my diabetes at the same time*,” and 50% endorsed “*I would rather be thin than have good control of my diabetes*,” reflecting a range of concerns related to the intersection between eating disorders and T1D.

## 4. Discussion

Despite the significant cardiovascular risks of comorbid T1D and excess weight in adolescents [5-7], there are few BLIs specific to youth with T1D. Analysis of qualitative interviews with adolescents with comorbid T1D and overweight/obesity, their caregivers, and pediatric endocrinologists found that participants were interested in a future BLI + T1D. Participants discussed preferred aspects of BLI + T1D that both align with and expand upon standard BLIs.

Youth who have a chronic disease, such as obesity or T1D, may feel isolated and be unaware of how many other peers can relate to them. While standard BLIs encourage social support in group settings [18-20], participants in the current study emphasized the necessity of having T1D-related social support. Participants described a desire for support-group elements (i.e., unstructured discussion time) beyond what is included in standard BLIs. This may be one reason why diabetes camp was brought up frequently during interviews. Diabetes camps offer youth with T1D an opportunity to connect with peers in the area, learn T1D disease management skills, and normalize having a chronic illness by providing an activity (i.e., camp) associated with typical-developing childhood life [46-48]. Though standard BLIs involve game-like activities to engage youth in weight management principles, increasing the amount of social discussion and games specific to T1D in a BLI + T1D may help to increase engagement and perceived social support amongst adolescents.

Participants suggested many program topics that are standard in a BLI including education regarding healthy eating, physical activity, and weight loss. A BLI + T1D will need to incorporate specific education of how to manage T1D while engaging in healthy eating, physical activity, and weight loss. Participants were interested in detailed tips on avoiding physical activity-related hypoglycemic events, diabetes-friendly meal/snack suggestions, and discussion of how engaging in these healthy lifestyle activities can improve HbA1C. While the idea of directly discussing weight was met with some hesitation from caregivers and endocrinologists, most participants agreed that the messaging around weight should be sensitive, reduce weight stigma, increase body esteem and self-confidence, and focus more on improving health as opposed to reducing the number on the scale, which is consistent with current adolescent BLIs [49].

Participants (including adolescents and caregivers) also wanted a BLI + T1D to involve attention to mental health. One-quarter of adolescents quantitively reported clinically significant levels of current diabetes-related distress. Diabetes burnout and diabetes-related distress amongst adolescents with T1D are highly related to glycemic stability, and diabetes burnout has been found to act as a barrier towards complying with T1D disease management recommendations [50, 51]. This is consistent with endocrinologists’ concern about overwhelming adolescents who have T1D by addressing both T1D and weight management. Psycho-education and skill building specific to diabetes-related distress may not only increase acceptability of a BLI + T1D but may also assist in improving glycemic stability. Furthermore, providing skills to manage T1D distress and burnout may decrease the risk of burnout across both T1D and excess adiposity. Caregivers and adolescents also wanted a BLI + T1D to involve topics that had nothing to do with diabetes at all in an effort to normalize having T1D. Future diabetes-related programs should consider including icebreaker activities, games, or arts and crafts that strengthen personal identity outside of T1D.

Most participants preferred an adolescent peer-group structure, with a health professional leading the group, and a parallel caregiver support group. Participants preferred groups to meet either in-person or a hybrid in-person/virtual model. In-person groups are standard for most evidence-based BLIs [17, 18]. However, the recent COVID-19 pandemic forced many programs to transition to virtual platforms. While virtual programming offers many advantages (e.g., reduces travel and time burden and widens participant geographic diversity), the results of the current study highlight that the adolescent participants in this study would likely be dissatisfied with virtual-only programming. Therefore, the results of the preferred program design align closely with established prepandemic BLIs.

Both qualitative and quantitative data aligned with the established literature regarding the high risk of disordered eating among adolescents with T1D [31, 33]. Clinically elevated DEPS-R scores found in the current study sample are equivalent to or slightly higher than that of other comparable adolescent samples [41, 52, 53]. One explanation may be that the adolescents in the current sample all met criteria for overweight or obesity. Research suggests a positive correlation between disordered eating behaviors and BMI [52, 53]. It was noted, however, that the DEPS-R questions may have less face validity for certain populations. Specifically, some of the questions that ask about weight cognitions may not correctly identify disordered eating symptoms in youth with overweight/obesity. The results of the current study highlight the importance of supporting adolescents with T1D and overweight/obesity with safe, evidence-based interventions rather than avoiding discussing weight loss all together. As shown in the results, left to their own devices, adolescents are more likely to engage in disordered eating behaviors to lose weight, as they lack the knowledge of healthy, effective skills. Evidence-based BLIs are associated with reductions in or no change in disordered eating symptoms [34]. It is critical that clinicians screen for disordered eating among their adolescent patients with comorbid T1D and elevated BMIs. It is also crucial that families and care providers acknowledge the very real cardiovascular risks associated with comorbid T1D and obesity and subsequent importance of addressing excess weight in youth with T1D.

### 4.1. Strengths and Limitations.

Persons with T1D, particularly youth, are often excluded from standard BLIs due to their complex disease management. This study provides results from patients that are often not represented in the weight management literature. Another strength of the study is the detailed data provided from qualitative interviews. As opposed to self-report measures, the research design allowed participants to provide varied perspectives and expand upon preferences. Alternatively, a relatively small sample size is a limitation of the present study. Though equivalent to other published qualitative literature in adolescence [26, 54], the data represent only a fragment of the population of adolescents with T1D. Another limitation of the study is that primary care physicians, diabetes educators, and dedicated weight management treatment providers were not recruited to participate in this study. Their insights could provide helpful suggestions for program development. The participants in this sample were all located in a similar area, which while beneficial for the design of a local BLI + T1D, may be missing important opinions from other geographic locations. Furthermore, this paper is missing important perspectives from a more diverse sample of adolescents with T1D and overweight/obesity. The literature on disordered eating and overweight/obesity among youth with T1D are limited. Future studies should consider evaluating these variables to provide further information on incidence, prevalence, and related outcomes. Future studies should also explore preferences for a BLI + T1D from a more racially, geographically, and economically diverse sample to verify the generalizability of the current study’s results.

The next step for this line of research is to use findings from the present study to adapt an evidence-based, adolescent BLI for teens with T1D. Future researchers, program developers, and clinicians working with patients with both obesity and T1D should consider some of the preferences described in this study to increase the acceptability of BLI-related healthcare provided to youth with T1D. These preferences include peer-social support, safe in-person contact with peers and healthcare providers, avoiding only content about T1D, and better integration of T1D disease management and healthy lifestyle behavior skill building. Given the recent AAP guidelines for treatment of adolescents with obesity highlighting bariatric surgery and pharmacotherapy treatment options in addition to BLIs, future research concerning other treatment options for adolescents with comorbid T1D and obesity are also needed. This is in line with AAP guidelines reflecting the need for future research on obesity treatment interventions for youth with medical comorbidities [22]. Clinicians should be screening for disordered eating behaviors amongst all their adolescents with T1D, especially those with overweight or obesity. While discussion of weight can be a sensitive topic for some, many adolescents with T1D are comfortable discussing weight and would prefer learning effective weight management skills than not to discuss weight at all. Trainings are available to clinicians to decrease weight bias and discuss weight with patients in a sensitive manner [55, 56].

## Figures and Tables

**Table 1:** Demographic characteristics.

Characteristics	*N* (or mean)	% (or *SD*)	Ranges
*Adolescent characteristics (n = 12)*			
Gender identity			
Female	7	58.3%	
Male	5	41.7%	
Age (yrs)	*M* = 14.83	*SD* = 1.19	13–17
Race			
White	11	91.7%	
Black or African American	1	8.3%	
BMI percentile for age and sex	*M* = 96.42	*SD* = 2.27	91–99
Overweight	2	16.67%	
Obesity	10	83.33%	
HbA1c	*M* = 7.86	*SD* = 0.88	6.4–9.2
*Caregiver characteristics (n = 12)*			
Gender			
Female	8	66.7%	
Male	4	33.3%	
Age (yrs)	*M* = 45.67	*SD* = 3.45	40–50
Race			
White	11	91.7%	
Black or African American	1	8.3%	
Household income (*n* = 11)	*M* = 124,272.73	*SD* = 82,869.89	24,000–300,000
*Highest level of caregiver education*			
High school degree or GED	1	8.3%	
Some college, no degree	4	33.3%	
Associates degree	1	8.3%	
Bachelor’s degree	2	16.7%	
Master’s degree	4	33.3%	
*Provider characteristics (n = 9)*			
Gender identity			
Female	4	44.4%	
Male	5	55.6%	
Age (yrs)	*M* = 44.89	*SD* = 9.05	34–62
Race			
White	8	88.9%	
Asian	1	11.1%	

**Table 2:** Exemplar quotes from the interviews.

Themes	Exemplar quotes
Program content	“*The program should address the challenges of losing weight with type one*, *such as how to exercise without having to take in a bunch of carbohydrates and how to adjust your insulin so you are less likely to go low. Including information and education on how food affects blood sugar so it can help them understand their diabetes better*.” (Endocrinologist, female) “…*how to cope in school when you start to feel high or low and it is hard to pay attention, and you have to eat a snack and everyone is paying attention to you*. … *Or when you are doing something active and have to sit out for something and everyone’s attention is on you*.” (15 years, female) “…*providing meal plans that a teen could follow would be beneficial, maybe something that she could do on her own*.” (Caregiver, female) “…*education about a well-balanced diet, how certain foods impact blood sugars*.” (Endocrinologist, female) “…*like let us forget about diabetes for a while and talk about other nondiabetes related things they have in common to show that diabetes does not define them*.” (Caregiver, male)
Programmatic messaging	“*(Weight) should definitely be included. People want to lose weight sometimes and people should have a safe way to do it because I have seen people try to starve themselves, which does not make you lose weight. It would be most beneficial for people instead of them trying to figure it out on their own*.” (17 years, male) “*It is important to consider (a) how they (families) perceive food and food consumption*, *(b) how they prepare their meals and what are the expectations from the family regarding food. These become important at times*.” (Endocrinologist, male) “*Making it very clear that we are not trying to cure your diabetes. We are trying to help you with it. We are trying to help you live with it. We are not trying to help you get rid of it. We are not trying to help you push it out the window. We are trying to help you live with it*.” (14 years, female) “*I think nowadays, it is very important, you know looking at the health side of it (weight and body image). Because you are hearing other kids, how they feel and you are hearing how their experience is*, *and how they have dealt with it or not dealt with it is very important to hear from a peer*, *not just a doctor or a parent or something*.” (Caregiver, male)
Program structure	“*I think, honestly, a combination of all of it would be useful so that it is a bit more convenient, but then definitely some face-to-face in-person because I just think that raises the level of accountability a bit when you actually have to go and kind of report what I have been doing*.” (Caregiver, female) “*I feel like at first, maybe like more frequently and then like once we get each other*… *and we feel like we are comfortable with each other*, *then*, *like we do not have to see each other as often*.” (15 years, female) “I*f you had a group of kids who you can relate to and you can talk to, you would be happier and healthier as a group of teenage type 1 diabetics. I think that would be a good thing*.” (17 years, male) “…*there are pros and cons to both. I think you can access more kids virtually*, *but I do not know if you can engage them as well*.” (Caregiver, male)
Social support	“*That is a big reason why my daughter goes to a diabetes camp. She feels like she belongs*.” (Caregiver, male) “N*ot talking about diabetes at a diabetes event*, *it makes you feel like I am just with some new people*.” (15 years, female) “*I think we are all at a different part in our journey. Some parents might not feel comfortable*, *you know*, *being that all-in parent and you know*, *but some might be like us*, *like*… *we want to be involved. But at this age, we do not want to*… *like we are walking on eggshells, but we still got to be in charge. And it is hard*.” (Caregiver, male)
Eating disorder risk	“*As much as I am worried for obese and overweight BMI*, *I am equally worried about omission of insulin to lose weight. You may need a more sensitive approach of doing weight management with T1D*.” (Endocrinologist, male) “…*the big issue is diabulimia. I do not know how to approach that*… *My educators are so afraid that it is happening that they are not willing to discuss the topic at all. You do not want to bring it up to someone who does not know about it*.” (Endocrinologist, male) “*Spinning it as a weight management thing would not be the way to go because I would worry very much about the diabulimia issue*.” (Endocrinologist, female) “*I always worry about hyper focusing on the weight. The number and the intake*… *because they have a different mechanism to demonstrate anorexic behaviors and bulimia behaviors. I think it needs to be dealt with very cautiously*.” (Endocrinologist, female)

## Data Availability

The data used to support the findings of this study are available upon request from the corresponding author.
